# Proof of Concept for Metacarpal Reconstruction Using a Custom 3-dimensional-Printed Titanium Implant: Design, Manufacturing, Surgical Technique, and Early Postoperative Outcomes

**DOI:** 10.1016/j.jhsg.2025.100874

**Published:** 2025-10-30

**Authors:** Ben Wesorick, Olabamibo Oke, Nadine Elizabeth Withrow, Daniel Herrera, Tara Mann, Lexi Lewis, Nur Nurbhai

**Affiliations:** ∗ restor3d, Durham, NC; † Weill Cornell Medical College, New York, NY; ‡ Division of Orthopedics, Department of Surgery, Medical College of Georgia, Augusta, GA; § Phoebe Orthopedics, Albany, GA; ‖ Orthopaedic Surgery, Medical College of Georgia, Augusta, GA

**Keywords:** 3-dimensional-printing, Additive manufacturing, Custom implant, Metacarpal reconstruction

## Abstract

This case report describes the use of a 3-dimensional-printed, custom titanium implant for the reconstruction of a complex post-traumatic metacarpal malunion. A 33-year-old woman with an open fracture of the right second metacarpal progressing to malunion from a motor vehicle accident underwent single-stage hand reconstruction using a custom implant. This report outlines preoperative planning, implant design, manufacturing, and surgical execution. Postoperative imaging confirmed appropriate implant positioning, soft tissue balancing, and deformity correction. The custom implant restored joint alignment and motion, resolving the preoperative fixed hyperextension contracture of the digit. At the 2-year follow-up, the implant demonstrated good incorporation without loosening and maintained digit function. This case demonstrates the unique application of a custom 3-dimensional-printed implant, from implant design through surgical execution, for complex hand reconstruction where alternative off-the-shelf options are not feasible, and highlights advantages in anatomical restoration and surgical precision in challenging patient populations.

Reconstruction using 3-dimensional-printed, customized implants offers a novel alternative to biologic reconstruction or amputation for complex bone deformities or large bone loss. Custom implants can restore anatomy altered by trauma, nonunions, malunions, infection, or malignancy when other treatments are inadequate.^1^ These implants are designed using CT imaging, additively manufactured with biocompatible materials, and accompanied by custom instrumentation to ensure precise surgical technique and implant placement.

Traditional reconstructive options, such as bone grafting, often require multiple surgical incisions for graft harvest, prolonged recovery, and higher complication rates.^2^ In contrast, custom 3-dimensional-printed implants may enable single-stage reconstruction, potentially reducing complications and improving outcomes.^2^

This case report illustrates the application of a custom 3-dimensional-printed implant in reconstructive hand surgery for a post-traumatic functionally limiting malunion and details the process of implant design, manufacturing, surgical technique, and postoperative clinical outcomes. Written informed consent was obtained from the patient for publication of this case report and images.

## Case Report

A 33-year-old woman with a history of smoking, diabetes, and hypertension sustained polytrauma following a motor vehicle accident, including an open fracture of the right second metacarpal. Initial management involved irrigation, debridement, laceration repair, and splinting. Nearly 3 years later, she presented with symptomatic malunion of the digit and extensor tendon scarring, causing fixed hyperextension deformity of the metacarpophalangeal (MCP) joint and loss of index finger flexion, severely limiting pinch and grasping function.

Because of the complex multiplanar deformity and bone loss of the index metacarpal, a conventional corrective osteotomy (eg, closing wedge or rotational step-cut) was not a viable solution without autologous bone grafting or a large corticocancellous bone graft, and the patient declined autograft harvest. Given the patient’s age, full-time employment, and overall health, a custom 3-dimensional-printed reconstruction was selected to optimize surgical outcomes.

### Design process

A high-resolution CT scan with submillimeter resolution (<1 mm slice thickness) of the affected and contralateral hands was obtained. Segmentation was completed in *Mimics* (Materialise NV), enabling the generation of digital 3-dimensional models of each bone that were then exported to *3-matic* (Materialise NV) for the digital implant and instrumentation design.

**Figure 1:**
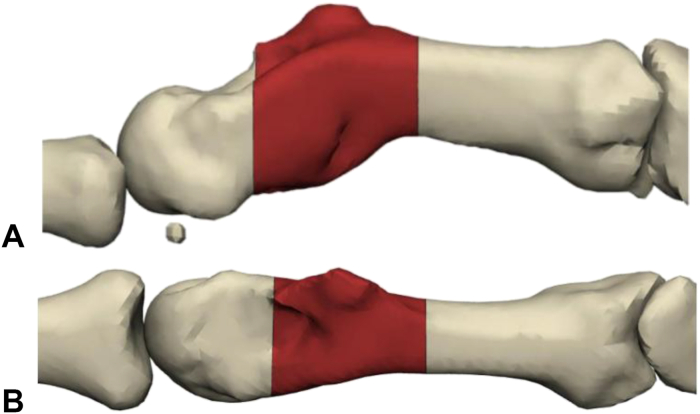
Lateral **A** and Anteroposterior **B** views of the planned resection of deformity from the contralateral mirror index metacarpal.

Anatomic correction and implant requirements were defined during a design meeting with the surgeon. The contralateral second metacarpal was mirrored and aligned with the proximal portion of the affected metacarpal using surface registration, serving as a reference for correcting length and rotational deformity. Proximal and distal resection margins were defined based on the malunited segment, with both planes positioned perpendicular to the intramedullary axis. Following the completion of virtual osteotomies, the distal bone fragment was rotated to match the contralateral anatomy, creating a segmental defect for reconstruction with the custom implant (Fig. 1A, B).

The implant was designed to span the segmental defect and replicate the external contours of the mirrored contralateral metacarpal, restoring normal anatomy and length. Porous titanium surfaces (Tidal Technology, restor3d) were incorporated at the proximal and distal interfaces to promote osseointegration.^3^ Proximal fixation included a 4.0 mm intramedullary stem and a transverse hole for a 1.5 mm bicortical interlocking screw. The implant was engineered to replicate physiologic bending and fatigue loading observed in metacarpals during benchtop studies. Bending strength exceeded that of traditional plate-and-screw constructs (246.1 N), whereas the 4.0-mm intramedullary component was optimized to withstand cyclic loads of 275 N·mm, based on the mechanical properties of 3-dimensional-printed titanium in tensile and fatigue loading.^4^^,^^5^ The distal end was rounded for intraoperative preparation with a matching MCP reamer, and two lag screws provided compression against the porous interfaces. To allow intraoperative flexibility, nominal and ±1 mm implant lengths were manufactured.

Custom metal guides included a cutting guide, a drill guide for stem preparation, and a final alignment guide. The cutting guide was designed to match preoperative anatomy and included three 1.1 mm K-wires, with outer K-wires serving as “joysticks” for fragment manipulation and alignment. The drill guide was designed to fit over the proximal K-wire to guide a 3.8 mm drill through the canal for a press-fit stem. The alignment guide was designed to slide over both joystick wires to align with the planned length and rotation. Finally, polymer trials were provided in three lengths to confirm intraoperative fit before implant insertion.

### Manufacturing

The implant was additively manufactured by restor3d using selective laser sintering of Ti-6Al-4V, a biocompatible titanium alloy with favorable mechanical properties. In selective laser sintering, a high-energy laser selectively fuses fine layers of titanium powder, building the implant layer by layer according to the digital model. This technique enables the simultaneous fabrication of both solid and porous features, allowing for a seamless integration between load-bearing and osseointegrative regions. The metallic surgical guides were fabricated in parallel using the same process.

Following printing, postprocessing included heat treatment to relieve residual stresses, reduce internal microvoids, and improve the fatigue resistance of the titanium alloy. Next, the implants underwent abrasive blasting to remove residual loose powder, and then, the solid implant surfaces were lightly polished to minimize the risk of irritation or abrasion to the surrounding soft tissues.

All polymer instruments were printed using *Formlabs Durable Resin* doped with barium sulfate to enhance radiopacity (restor3d). The polymer components were produced via stereolithography and a photocuring process to fully polymerize any residual uncured resin. The radiopacity allowed the guides and trial implants to be visualized intraoperatively under fluoroscopy, supporting accurate placement and alignment.

After passing the final dimensional and visual inspection, the implants and instrumentation were sterilized at the hospital prior to surgery.

### Surgical technique

**Figure 2:**
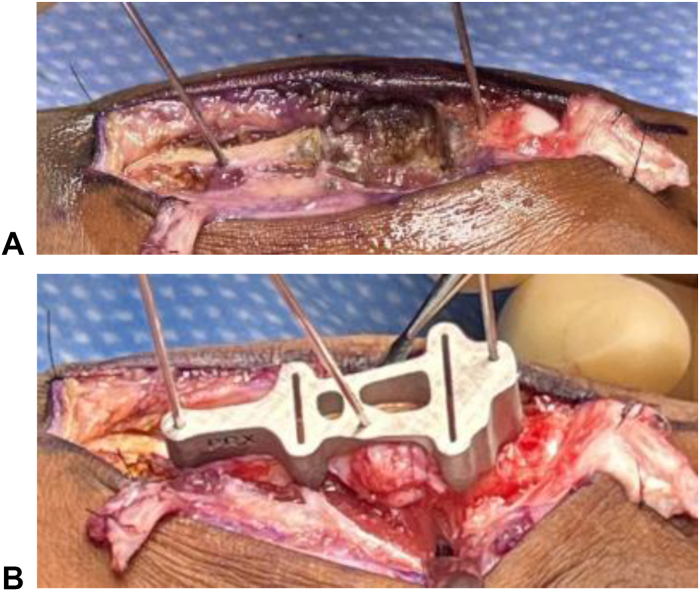
Images of the initial injury **A** and custom metallic cutting guide **B** used to skeletonize the bone in preparation for excision.

The patient was positioned supine with regional anesthesia and tourniquet control. A curvilinear dorsal incision from the MCP joint to the metacarpal base incorporated the prior laceration scar. Full-thickness skin flaps were elevated, and dissection proceeded to the extensor tendons. The extensor digitorum communis tendon was severely fibrotic and adhered centrally but intact; no significant scarring of the extensor indicis proprius tendon. The extensor digitorum communis tendon was carefully divided to expose the malunited metacarpal, which was skeletonized. Osteotomies were performed using the custom cutting guide (Fig. 2A, B), excising the malunited segment. The intramedullary canals were reamed, and retained K-wires were used as joysticks for alignment.

**Figure 3:**
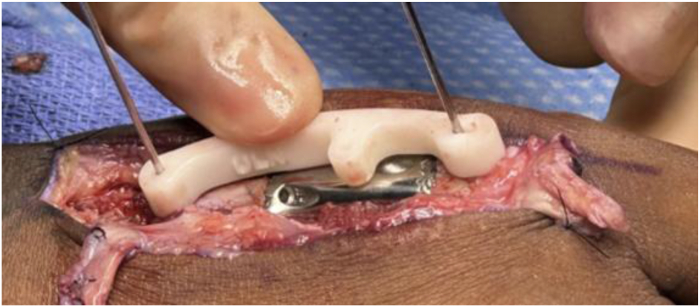
Distraction and alignment guide placed over the joystick wires.

**Figure 4:**
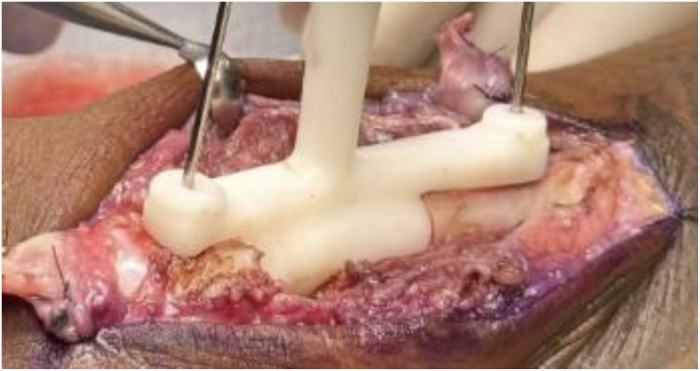
Example of trial implants (provided in three lengths) to allow intraoperative flexibility.

**Figure 5:**
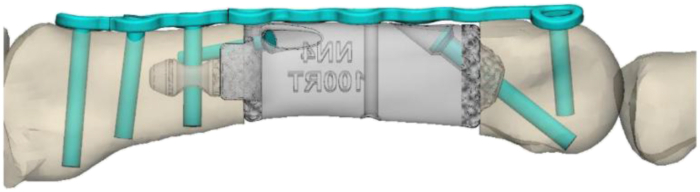
One 1.5 mm interlocking bicortical screw through the transverse hole and two 1.5 mm lag screws proximally and distally for fixation.

**Figure 6:**
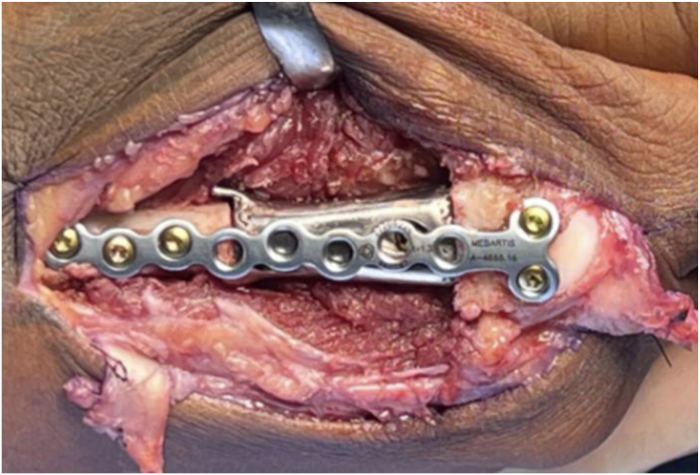
Dorsal MedArtist 2.0 mm plate used to augment fixation.

The distraction and alignment guide (Fig. 3) was placed to reproduce the planned correction. Then, the distal fragment was reamed using a matching MCP reamer to create a congruent contact surface for the distal implant interface. Trial implants in three lengths (Fig. 4) were sequentially inserted and assessed under fluoroscopy for length restoration and alignment. Upon confirming optimal fit, the selected implant was press-fit into the proximal canal, with the distal porous end seated into the reamed distal segment. Fixation was achieved using one 1.5 mm interlocking bicortical screw through the transverse hole and two lag screws proximally and distally to compress the bone onto the porous surfaces (Fig. 5). A dorsal MedArtist 2.0 mm plate was applied to augment fixation, with screws placed around the implant (Fig. 6).

Fluoroscopic imaging confirmed implant positioning. To address the extensor tendon length discrepancy, a gracilis allograft intercalary graft was used for reconstruction. The graft was tensioned with the MCP joint held in slight flexion to optimize functional arc and soft tissue balance. After irrigation, the wound was closed in layers; estimated blood loss was 30 cc. The index finger hyperextension deformity was corrected, the MCP joint range of motion was satisfactory without scissoring, and a volar splint was applied to maintain neutral extension of wrists and digits.

### Postoperative follow-up

**Figure 7:**
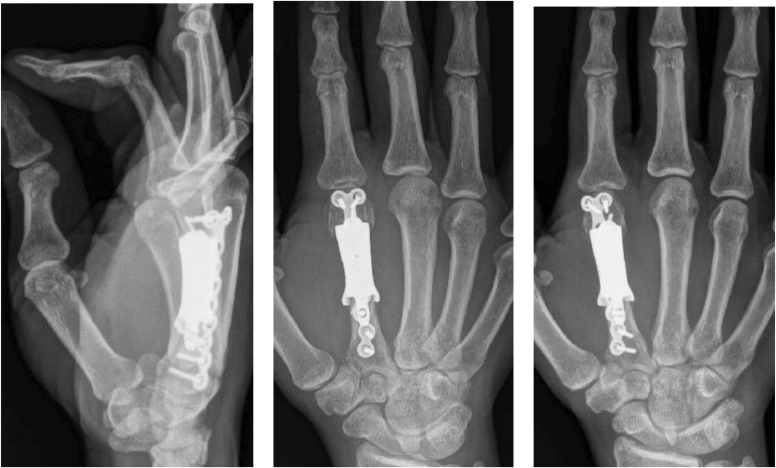
Follow-up of index metacarpal reconstruction with 3-dimensional-printed prosthesis demonstrates good incorporation and osseointegration of the prosthesis to the mating surfaces of the metacarpal without loosening.

The patient remained adherent to follow-up through 8 weeks after surgery with no complications (Fig. 7). At 2 years, the incision was well-healed with no signs of infection, 80° MCP flexion, full proximal interphalangeal and distal interphalangeal joint motion, and good pinch and grasp function. She reported no pain or functional limitations and had resumed daily activities, indicating a satisfactory outcome.

## Discussion

This case demonstrates successful single-stage correction of a complex metacarpal malunion using a custom 3-dimensional-printed implant, with early results showing deformity correction and satisfactory motion. Although effective, established osteotomy techniques merit consideration: step-cut osteotomy offers multiplanar correction with stability and high union rates,closing wedge osteotomy offers technical simplicity but shortens the bone, and opening wedge techniques preserve length but may require grafting.^6^, ^7^, ^8^, ^9^, ^10^

Unique challenges in this case included multiplanar deformity, bone loss, poor bone quality, and autograft refusal. Conventional osteotomies risked further shortening or unreliable union, particularly given comorbidities (smoking, diabetes). Although additive manufacturing comes with risks (incomplete fusion, mechanical failure, foreign body reactions, infection), mechanical stability and biological fixation were achieved using mirrored contralateral anatomy and custom guides for precision.

Limitations include single-case data, unknown long-term outcomes, and the cost and logistics of custom fabrication. We acknowledge concerns regarding long-term implant durability, particularly fatigue failure through cantilever bending in younger patients; however, our design incorporated fatigue resistance (clinical validation is pending). This treatment approach balanced immediate functional restoration against potential future revision requirements, with comprehensive informed consent regarding these uncertainties.

Despite these challenges, additive manufacturing enables precise, single-stage reconstruction and holds significant promise for restoring function in intricate skeletal structures. Further research is warranted to assess durability and reproducibility.

## Conflicts of Interest

Mr Wesorick, Ms Lewis, and Dr Mann are paid employees of restor3d, Inc. and have received a portion of their compensation in the form of equity. Dr Nurbhai has equity as an investor in restor3d, Inc. No benefits in any form have been received or will be received by the other authors related directly to this article.
